# Markers of increased atherosclerotic risk in patients with chronic kidney disease: a preliminary study

**DOI:** 10.1186/s12944-016-0191-x

**Published:** 2016-02-03

**Authors:** Anna Gluba-Brzózka, Marta Michalska-Kasiczak, Beata Franczyk, Marek Nocuń, Peter Toth, Maciej Banach, Jacek Rysz

**Affiliations:** Department of Nephrology, Hypertension and Family Medicine, WAM University Hospital of Lodz, Poland, Żeromskiego 113, 90-549 Łódź, Poland; Department of Hypertension, Medical University of Lodz, Poland, Żeromskiego 113, 90-549 Łódź, Poland; Nofer Institute of Occupational Medicine, Lodz, Poland, Św. Teresy od Dzieciątka Jezus 8, 91-348 Łódź, Poland; Preventive Cardiology, CGH Medical Center, Sterling, IL USA; The Johns Hopkins Ciccarone Center for the Prevention of Heart Disease, Baltimore, MD USA; Healthy Aging Research Center, Medical University of Lodz, Lodz, Poland

## Abstract

**Background:**

The prevalence of chronic kidney disease is rising continuously. Cardiovascular disease is among leading causes of death and premature mortality of patients with chronic kidney disease. Even the earliest stages of chronic kidney disease are associated with higher risk of subsequent coronary heart disease. The aim of this study was to determine markers of increased risk of atherosclerosis in CKD.

**Methods:**

The study group consisted of a total of 80 patients (20 patients with stage I/II CKD, 20 with stage III CKD, 20 stage IV CKD and 20 stage V/dialysis) and 24 healthy volunteers. Levels of proteins (osteoprotegerin, osteopontin, osteocalcin, matrix γ-carboxyglutamic acid protein, fetuin A, MMP-2, MMP-9, TIMP-1, TIMP-2) and biochemical parameters were measured to analyse their influence on atherosclerosis risk in CKD patients. Cardiac echocardiography was performed to assess structural integrity and function, presence of left ventricular hypertrophy and systolic and diastolic function dysfunction.

**Results:**

This study shows that the prevalence of ventricular hypertrophy (95.3 %) and diastolic dysfunction (93.2 %) in CKD patients is high. Also E/E’ ratio was significantly higher (13.6 ± 4.4, *p* = 0.001), tricuspid insufficiency (27.3 in CKD I/II vs. 71.4 in CKD V, *p* = 0.016), contractile dysfunction (33.3 in CKD I/II vs. 78.9 in CKD V, *p* = 0.040), mitral valve calcification (0 in CKD I/II vs. 28.6 in CKD V, *p* = 0.044) and aortic valve calcification (0 in CKD I/II vs. 61.9 in CKD V, *p* = 0.0008) were significantly more frequent in patients with CKD stage V/dialysis than in other groups. Only MMP-2, MMP-2/TIMP-2 ratio and TIMP-1 differed significantly between groups.

**Conclusions:**

This study shows high prevalence of ventricular hypertrophy and diastolic dysfunction in CKD patients. Contractile dysfunction, mitral and aortic valve calcification in HD patients were significantly more frequent than in patients with other CKD stages. Significantly increased levels of MMP-2, MMP-2/TIMP-2 ratio and lower TIMP-1 suggests that these factors may be involved in the pathogenesis of atherosclerosis in CKD patients.

## Background

Chronic kidney disease (CKD) is defined by KDIGO in Clinical Practice Guideline for the Evaluation and Management of Chronic Kidney Disease published in 2013 as abnormalities of kidney structure or function, present for over 3 months, with implications for health and CKD is classified based on cause, GFR category, and albuminuria category (CGA) [1]. The prevalence of chronic kidney disease is rising continuously. According to National Kidney Foundation (NKF) KDOQI guidelines, chronic kidney disease, irrespective of diagnosis, is associated with increased risk of cardiovascular disease (CAD), including coronary heart disease, cerebrovascular disease, peripheral vascular disease, and heart failure, due to both ‘traditional’ (defined in the Framingham Heart Study) and ‘chronic kidney disease related’ CAD risk factors, and, thus, these patients have risk on par with the highest CAD risk group [2]. The prevalence of uraemia-related (non-traditional) factors increases along with the decline in kidney function. Cardiovascular disease is one of the leading causes of death and premature mortality of patients with chronic kidney disease. According to recent studies, even the earliest stages of chronic kidney disease are associated with higher risk of subsequent coronary heart disease [3, 4]. It has been suggested that the assessment of CKD-associated CAD risk factors together with conventional risk factors should be performed in order to improve the prediction of coronary heart disease risk [2]. Moreover, patients with manifestations of cardiovascular disease should be screened for evidence of kidney disease [3, 5, 6]. The reduction in risk factors seems to be effective in lowering cardiovascular morbidity and mortality in patients with CKD [2]. According to the report of NKF Task Force on Cardiovascular Disease in Chronic Renal Disease, the mortality due to CVD was 10 to 30 times higher in dialysis patients than in the general population despite stratification for sex, race, and the presence of diabetes [7]. CVD mortality in dialysis patients remained ~5-fold higher than in the general population after stratification for age [8]. In patients with CKD the prevalence of arteriosclerosis (remodelling of large arteries) and cardiomyopathy is higher than in general population [9]. A high prevalence of a proinflammatory state, endothelial dysfunction, hypertension, and dyslipidemia associated with renal disease may explain the acceleration of atherosclerosis with a high prevalence of coronary ischemia and CV events in CKD. However, the exact mechanisms of atherosclerotic and arteriosclerotic changes in the setting of CKD formation are not yet fully characterized.

### Aim

The aim of this study was to determine markers of increased risk of atherosclerosis in CKD.

## Methods

The study group consisted of a total of 80 patients (20 patients with stage I/II CKD, 20 with stage III CKD, 20 stage IV CKD and 20 stage V/dialysis) hospitalized in the Department of Nephrology, Hypertension and Family Medicine. The control group consisted of 24 volunteers without CKD, recruited among patients hospitalized due to causes other than CAD, tumours or diabetes mellitus. All persons involved in this study signed informed a consent form before the collection of blood samples. The purpose and methodology of this study was approved by the Bioethics Committee of the Medical University of Lodz (no. RNN/79/12/KB). Total cholesterol, low-density lipoprotein (LDL), high-density lipoprotein (HDL), triglyceride (TG), albuminuria, serum calcium and phosphate, Fe, total iron-binding capacity (TIBC), C-reactive protein (CRP), alkaline phosphatase activity, creatinine, urea, uric acid, total protein, the level of fibrinogen and D-dimer were also determined. In addition, cardiac echocardiography was performed to assess structural integrity and function, the presence of left ventricular hypertrophy and systolic and diastolic function dysfunction. Contractility disorder was diagnosed when ejection fraction (EF) is below 44 %. The E/A is defined as a ratio of the early (E) to late (A) ventricular filling velocities, while E/E’ is a ratio of early filling (E) and early diastolic mitral annular velocity (E’) [10]. The levels of studied proteins and biochemical markers were analysed in blood of all people involved in the study. The study excluded patients with diagnosed cancer and advanced cardiovascular disease. In the present study, concentrations of proteins involved in the processes of vessel wall calcification and bone metabolism disorders (osteoprotegerin [TECOMedical, no. 8034], osteopontin [RayBiotech, ELH-OPN-001], osteocalcin [TECOMedical, no. 8002], matrix γ-carboxyglutamic acid protein (MGP) [USCN Life Science, E91477Hu], fetuin A [TECOMedical, no. KT-800]) and vascular remodelling (MMP-2, MMP-9, TIMP-1, TIMP-2 [Raybiotech: ELH-MMP2-001, ELH-MMP9-001, ELH-TIMP1-001, ELH-TIMP2-001]) were measured in order to analyse their influence on atherosclerosis risk in CKD patients. Levels of these proteins were determined by the ELISA method according to the manufacturer’s instructions. Estimated glomerular filtration rate (GFR-MDRD) was calculated using the Modification of Diet in Renal Disease:

GFR (ml/min/1.73 m^2^) = 186 x (Creat/88.4)^− 1.154^x (Age)^− 0.203^x (0.742 if female) x (1.210 if black), and the classification into CKD stage confirmed using the CKD-EPI equation:

$$ \mathrm{G}\mathrm{F}\mathrm{R} = 141\ \mathrm{x}\  \min {\left({\mathrm{S}}_{\mathrm{cr}}/\upkappa \mathrm{o}\mathrm{r}\ 1\right)}^{\upalpha}\mathrm{x}\  \max {\left({\mathrm{S}}_{\mathrm{cr}}/\upkappa \mathrm{o}\mathrm{r}\ 1\right)}^{-1.209}\mathrm{x}\ 0.{993}^{\mathrm{Age}}\mathrm{x}\ \left(1.018\ \mathrm{if}\ \mathrm{female}\right)\ \mathrm{x}\ \left(1.159\ \mathrm{if}\ \mathrm{black}\right), $$

where:

S_cr_ - serum creatinine (mg/dL), κ - 0.7 for females and 0.9 for males, α is -0.329 for females and -0.411 for males [74].

Creatinine level was measured with enzymatic method.

This work was funded by Iuventus Plus 2010 grant no. IP2010009870 from the Polish Ministry of Science and Higher Education.

### Statistical analysis

Results were expressed as mean with standard deviation (mean ± SD) for continuous variables with normal distribution or as a median with interquartile range (median, 25 %-75 %) in all other cases. Categorical variables are presented as percentages related to the size of the study group. Shapiro-Wilk test was used to verify normal distribution of variables and Levene test to analyse the homogeneity of variance. Standard Student *t* test was used for the comparison of data showing no departures from normality and for multiple comparisons (more than two groups) one-way ANOVA with post hoc Scheffe tests was used. If at least one of the aforementioned criteria is not met, non-parametric Mann–Whitney *U* test and detailed or non-parametric analysis of variance (Kruskal-Wallis test) with post hoc Connover-Inman tests was used, respectively. The *χ*^2^ test of independence was used for the analysis of discontinuous variables. The analysis of logistic regression was used for the analysis of relationship between the occurrence of cardiovascular disorders and CKD, age, sex, and the concentration of selected proteins. All the echocardiographic images were analysed by the single investigator and repeated in order to assess the intra-observer variability. Intra-observer variability of echocardiographic parameters was determined on the basis of the intra-class correlation coefficient (ICC) with 95 % CI. A value of *p* < 0.05 was considered significant. Calculations were made with the use of statistical R program [11].

## Results

Eighty patients in the study group with the average age of 67.2 ± 11.7 and 24 patients in control group with the age of 61.2 ± 9.6 (*P* = 0.042) were involved in the study. There were 45 men and 35 women (56.2 % and 43.8 %, respectively) in the study group and 7 men and 17 women (29.2 % and 70.8 %) in the control group (*P* = 0.01). Hypertension occurred significantly more often in the study group (88.3 % vs. 37.5 %, *p* < 0.0001).

The analysis of biochemical parameters in both groups revealed in patients with CKD stages I-V statistically lower concentration of Na^+^ (137.9 ± 3.4 vs. 140.1 ± 2.8, *P* = 0.006), haemoglobin level (11.7 ± 1.9 vs. 12.8 ± 1.4, *P* <0.01), iron (12.1 ± 5.6 vs. 18.1 ± 7.0, *P* = 0.001), and higher level of hsCRP (14.7 ± 30.7 vs. 3.9 ± 6.6, *P* < 0.005), inorganic phosphate (1.36 ± 0.44 vs. 1.14 ± 0.15, *P* = 0.001), and triglycerides (1.88 ± 1.07 vs. 1.46 ± 0.62, *P* = 0.02), in comparison with the control group. Moreover, in the study group, levels of markers of renal function, such as urea (14.0 ± 8.4 vs. 5.5 ± 1.9, *P* <0.0001), creatinine (276.2 ± 217.4 vs. 80.1 ± 11.3, *P* <0.0001) and uric acid (386.7 ± 135.9 vs. 271.7 ± 53.0, *P* <0.0001) were also significantly increased. In patients with chronic kidney disease, GFR-MDRD was significantly lower in comparison to the control group (35.0 ± 24.5 vs. 86.4 ± 16.4). The prevalence of comorbidities and frequency of used drugs differed significantly between control and study groups. Baseline characteristics of enrolled patients is summarized in Table 1.

**Table 1:** Baseline characteristics of enrolled patients

Table 1	CKD Stages I-V	Control group	*p*
	*N*=80	*N*=24	
Age	67.2 ± 11.7	61.2 ± 9.6	0.042
Gender (males %)	56.2%	29.2%	0.01
Diabetes mellitus	33.3%	0%	0.0022
Atrial fibrillation	20.83%	13.79%	NS
Hypertension	88.3%	37.5%	<0.0001
Lipid disorders	30.6%	41.4%	0.039
Heart failure	63.9%	0%	<0.0001
Hypertensive nephropathy	5.6%	0%	NS
Diabetic nephropathy	4.2%	0%	NS
Beta-blockers	61%	20,8%	<0.007
CA-blockers	40.5%	8.3%	0.004
ACE inhibitors	69.6%	41%	0.011
Diuretics	90.5%	16.7%	<0.0001
Statins	97.5%	43%	<0.0001
Erythropoietin^a^	5.6%^a^	0%	<0.0001
Mean dialysis vintage [months]^a^	27±9^a^	0	<0.0001

^a^ applies only to CKD stage V patients

Echocardiographic examination results are summarized in Table 2.

**Table 2:** The summary of echocardiographic examination results

Table 2	Control group	CKD Stages I-V	*p*
	*N*=24	*N*=80	
E/A	0.8 (0.65-1.1)	0.9 (0.8-1.2)	NS
E/E’	7.5±2.1	9.9±4.7	NS
Hypertrophy [%]	52	95.3	NS
Diastolic dysfunction [%]	29	93.2	NS
Contractility dysfunction [%]	57.1	52.8	NS
Stenosis	0	3.2	NS
Mitral valve fibrosis	53.3	81.3	*P*<0.01
Aortic valve fibrosis	0	14.1	NS
Mitral valve calcification	13.3	14.1	NS
Aortic valve calcification	0	30.2	*P*<0.02
IM	46.7	63.1	NS
IA	0	6.7	NS
IT	16.7	43.8	NS

Abbreviations used in Table 1: *IM* mitral insufficiency, *IA* atrial insufficiency, *IT* tricuspid insufficiency, *NS* not significant

All the echocardiographic measurements were performed by the same person. The intra-observer variability by ICC (interclass correlation coefficient) varied from 0.82 to 0.96. Echocardiographic examination revealed significant differences only in the occurrence of mitral valve fibrosis and aortic valve calcification between the study and control groups. More significant differences in echocardiographic results were observed when each CKD stage was analysed separately. The results of this analysis are presented below, in Table 3.

**Table 3:** The summary of echocardiographic examination results of patients divided into CKD stage groups

Table 3.	Stage I/II	Stage III	Stage IV	Stage V	*p*
	*N*=20	*N*=20	*N*=20	*N*=20	
E/A	0.8 (0.8-1.1)	0.8 (0.7-0.95)	0.9 (0.8-1.2)	0.9 (0.8-1.3)	NS
E/E’	7.4±2.0	8.1±3.9	7.1±3.5	13.6±4.4^1,3,6^	*P*=0.001
Diastolic dysfunction [%]	81.8	93.3	100.0	94.7	NS
Contractility disorders [%]	33.3	42.9	36.4	78.9^1,5^	*P*=0.040
Mitral valve fibrosis [%]	81.8	80.0	70.6	90.5	NS
Aortic valve fibrosis [%]	9.1	20.0	23.5	4.8	NS
Mitral valve calcifications [%]	0	0	17.6	28.6^1^	*P*=0.044
Aortic valve calcifications [%]	0	13.3	23.5	61.9^2,5^	*P*=0.0008
IM [%]	54.5	50.0	58.8	81.0	NS
IA [%]	10.0	12.5	0	4.8	NS
IT [%]	27.3	25.0	37.5	71.4^1^	*P*=0.016

^1^ *p*<0.05; ^2^ *p*<0.01 vs Stage I; ^3^ *p*<0.05; ^4^ *p*<0.01 vs Stage III; ^5^ *P*<0.05; ^6^ *p*<0.01 vs Stage IV

This analysis revealed significant differences in E/E’, presence of contractility disorders, occurrence of mitral and aortic valve calcifications and tricuspid insufficiency.

Our analysis of the relationship between selected proteins and CKD stage are demonstrated in Table 4.

**Table 4:** The results of analysis of selected proteins concentrations

Table 4	Control group	Stage I/II	Stage III	Stage IV	Stage V	p
	*N*=24	*N*=20	*N*=20	*N*=20	*N*=20	
Fetuin A [ng/ml]	102,9±61,1	110,6±74,7	120,1±82,2	102,1±77,4	125,2±63,3	NS
MMP-2 [ng/ml]	103,0±55,7	161,6±105,7	213,0±187,8	270,9±144,5**	228,5±117,6*	*P*=0,002
MMP-9 [ng/ml]	18,4±5,6	16,9±6,5	17,4±5,9	18,0±6,5	18,0±7,6	NS
TIMP-1 [ng/ml]	23,6±2,9	19,9±2,5**	22,1±2,7	21,4±2,5	21,1±1,9*	*P*=0,002
TIMP-2 [ng/ml]	21,0±2,8	21,8±2,2	22,9±1,4*	21,5±2,0	22,0±1,7	NS
MMP-2/TIMP-2	4,7±2,9	7,4±4,6	9,2±7,9	11,9±6,9**	10,5±5,8*	*P*=0,002
MMP-9/TIMP-1	0,93±0,43	0,80±36	0,77±0,26	0,81±0,39	0,83±0,39	NS
Osteocalcin [ng/ml]	6,2±4,0	7,8±4,5	7,0±3,8	5,0±4,4	3,9±4,1	NS
Osteopontin [ng/ml]	38,0±22,5	11,3±5,1	29,7±33,6	29,8±29,9	28,7±19,7	NS
Osteoprotegerin [pmol/l]	6,0±5,3	4,5±5,6	6,3±5,4	6,4±5,1	8,7±6,1	NS
MGP [ng/ml]	103,7±30,4	105,8±44,4	103,5±29,5	100,7±41,9	88,7±55,0	NS

*p<0.05; **p<0.01 vs control

The analysis of the concentrations of proteins associated with bone metabolism (fetuin A, osteocalcin, osteopontin, osteoprotegerin and MPG) revealed no statistically significant differences between the control group and patients with chronic renal failure. It was observed that the concentration of osteocalcin was highest in patients with stage I/II CKD and gradually decreased to its lowest value in patients with stage V/dialysis. Similar proportional decreases through CKD stages were observed with osteocalcin and MPG. Osteoprotegerin concentration was lowest in subjects with stage I/II CKD and gradually increased to reach its highest value in patients with stage V/dialysis. However, these trends were not statistically significant which may be related to the small size of each group.

Statistically higher levels of MMP-2 in patients with chronic kidney disease are observed as compared to the control group (*p* = 0.002) were observed in this study (Table 4). The lowest concentration of MMP-2 was seen in patients with CKD stage I/II and the highest in patients with stage IV and in those on dialysis. Significant differences in serum concentrations of metalloproteinase inhibitor TIMP-1 (*p* = 0.002) and MMP-2/TIMP-2 ratio were also observed. Statistically significant results of multivariable analysis are presented in Table 5.

**Table 5:** Multivariate analysis of obtained results

Table 5	*p*	OR	95.0 % CI range
The presence of heart failure			
Chronic kidney disease	0,030	4,625	(1,161 - 18,429)
Osteopontin	0,045	0,979	(0.960 - 1,000)
Age	0,010	1,074	(1,017 - 1,135)
(independent of osteocalcin, osteoprotegerin, fetuin, gender)			
Age	0,004	1,083	(1,026 - 1,142)
MMP-2	0,048	1,004	(1,000 - 1,009)
(independent of gender, chronic kidney disease, MMP-9, TIMP-1, TIMP-2)			
Age	0,001	1,109	(1,046 - 1,176)
Chronic kidney disease	0,038	5,361	(1,098 - 26,176)
(independent of gender, MGP, ANGII, chronic kidney disease)			
Age	0,004	1,083	(1,026 - 1,143)
Chronic kidney disease	0,013	7,449	(1,532 - 36,222)
AIP	0,053	8,694	(0,977 - 77,396)
(independent of gender, ABCA1, ABCG1, non-HDL cholesterol, urea)			
Phosphates above normal level			
Osteoprotegerin	0,025	1,163	(1,019 - 1,328)
(independent of osteocalcin, osteopontin, fetuin, age, gender)			
Presence of hypertension			
Chronic kidney disease	0,007	8,202	(1,790 - 37,584)
(independent of age, gender, MMP-2, MMP-9, TIMP-1, TIMP-2)			
The presence of atherosclerosis			
Gender	0,017	0,223	(0,065 - 0,769)
(independent of age, chronic kidney disease, MMP-2, MMP-9, TIMP-1, TIMP-2)			
Gender	0,045	0,301	(0,093 - 0,971)
(independent of age, chronic kidney disease, MGP, GM-CSF, ANGII			
Lipid disorders			
Non-HDL cholesterol	0,007	1,881	(1,186 - 2,983)
(independent of age, gender, chronic kidney disease, ABCA1, ABCG1, AIP, urea)			
AIP – high risk			
TCh	0,000	0,019	0,003 – 0,138
Non-HDL cholesterol	0,000	110,134	11,416 - 1062,535
(independent of age, gender, chronic kidney disease, ABCA1, ABCG1, urea)			

Abbreviations: *MMP-2* matrix metalloproteinase 2, *MMP-9* matrix metalloproteinase 9, *TIMP-1 & TIMP-2* tissue inhibitor of metalloproteinases-1&2, *MGP* matrix Gla protein, *AngII* angiotensin II, *GM-CSF* granulocyte-macrophage colony-stimulating factor, *AIP* atherogenic index of plasma, *TCh* total cholesterol, *ABCA1* ATP binding cassette subfamily A member 1, *ABCG1* ATP-binding cassette sub-family G member 1

In this analysis, the presence of heart failure was associated with the presence of chronic kidney disease, the level of osteopontin, age, MMP-2 and AIP and this relationship was independent of osteocalcin, osteoprotegerin, fetuin, gender, MMP-9, TIMP-1, TIMP-2, MGP, ANGII, ABCA1, ABCG1, non-HDL cholesterol and urea).

There was also relationship between phosphates above normal level and osteoprotegerin. Moreover, the presence of hypertension was associated with chronic kidney disease, the presence of atherosclerosis associated only with gender, while lipid disorders with non-HDL cholesterol, AIP (high risk) and total cholesterol. An analysis of the relationship between protein concentrations and various biochemical markers was also performed. Statistically significant results of this analysis are presented in Table 6.

**Table 6:** The relationship between selected protein concentration and other parameters

Table 6.	Hypertension	ALP ↑	CRP ↑	Fe ↓	↑ inorganic P	↑ Uric acid	↑ Urea
Fetuin A [ng/ml]	112.1±74.3	168.9±65.7^4^	108.7±74.5	115.6±77.3	106.4±69.2	114.1±83.3	120.7±73.6
MMP-2 [ng/ml]	217.8±150.9^2^	225.4±82.0	229.4±116.1^5^	232.1±158.9	240.2±134.0	264.1±163.8^1^	227.7±132.8^4^
MMP-9 [ng/ml]	18.3±6.5	17.9±7.2	19.0±7.0	17.8±6.3	17.4±6.9	19.6±5.6^4^	18.0±7.0
TIMP-1 [ng/ml]	21.5±2.7	21.1±2.2	21.4±2.2	21.8±2.4	21.3±2.1	20.9±2.5^4^	21.5±2.4
MMP-2/TIMP-2	9.8±6.8^3^	10.4±3.4	10.6±5.6^4^	10.5±6.8	11.2±6.6	11.6±7.4^1^	10.3±6.2^4^
MMP-9/TIMP-1	0.84±0.35	0.86±0.41	0.88±0.39	0.83±0.33	0.82±0.37	0.91±0.31^6^	0.83±0.41
Osteocalcin [ng/ml]	5.9±4.1	3.2±3.2	4.9±4.3^5^	5.0±4.7	4.1±3.6^4^	5.6±3.6	4.9±4.1^1^
Osteopontin [ng/ml]	26.3±26.1^3^	17.6±7.7	25.9±20.9	32.4±31.1	31.4±25.1	26.4±30.0	28.0±23.3
Osteoprotegerin [pmol/l]	7.3±5.8^1^	8.2±4.3	7.2±4.9	7.9±5.8^4^	9.5±6.4^1^	7.4±5.7	7.3±5.6
MGP [ng/ml]	103.7±30.4	62.0±28.4^4^	93.2±44.2	85.8±39.2^1^	97.4±58.6	102.1±38.4	95.7±43.4

^1^ *p*<0.01; ^2^ *p*<0.0001; ^3^ *p*=0.065; ^4^ *p*<0.05; ^5^ *p*<0.07; ^6^ *p*<0.08

## Discussion

This preliminary study analysed the possible markers of atherosclerotic and calcification processes occurring in CKD patients and possible novel mechanisms of increased cardiovascular risk in this group of patients.

The results of biochemical parameters analysis (significantly lower levels of Na^+^, haemoglobin and iron, as well as higher levels of CRP, PO_4_^−^ and triglycerides in patients with CKD stages I-V in comparison with the control group) are consistent with results of other studies [12, 13] and are associated with kidney damage. Also, significantly higher concentrations of urea, creatinine and uric acid in patients with CKD stages I-V are not surprising, since these are established markers of kidney function.

This study shows that the prevalence of ventricular hypertrophy (95.3 %) and diastolic dysfunction (93.2 %) in CKD patients is high. According to the literature, left ventricular hypertrophy appears in approximately 40 % of patients with chronic renal insufficiency, and is even more frequent (75 %) at the onset of ESRD [14, 15]. Progressive left ventricular enlargement is considered as the most typical morphological pattern of dialysis patients and it is a crucial prognostic factor for cardiovascular mortality in ESRD patients [16, 17]. Diastolic dysfunction, which is frequent in chronic kidney disease (CKD) patients, accounts for 40 %-66 % of cardiovascular complications [18]. However, there is still a controversy concerning which parameter (E/A, E’, E/E’) is of better predictive and prognostic value for the diagnosis of diastolic dysfunction and the assessment of its clinical outcomes [19]. It has been suggested that the use of multiple echocardiographic indices to diagnose and to grade diastolic dysfunction seems to be the best solution. This analysis revealed that in patients with CKD stage V/dialysis the E/E’ ratio was significantly higher than in other groups (13.6 ± 4.4, *p* = 0.001) and that in this group of patients tricuspid insufficiency was significantly more prevalent (*p* = 0.016). The results of the de Bie et al. [20] study confirm that diastolic dysfunction is highly prevalent among dialysis patients but they imply that its prevalence in this group of patients might be underestimated using conventional measurements. The diagnosis of LV diastolic dysfunction has been demonstrated to provide independent, prognostic value for long-term mortality and cardiovascular death in patients with end-stage renal disease [21]. As shown by Han et al. [19], the increase in E/E’ (E/E’ > 15) and left atrium (LA) volume index (LAVI > 32 mL/m^2^) are significant risk factors for CV events in incident dialysis patients with preserved LV systolic function.

Vascular calcification (VC) within the media and intima layers of arteries contributes considerably to the greater mortality of patients with chronic kidney disease [22, 23]. This pathological calcification seems to be associated with an elevated serum calcium phosphate and with differentiation of vascular or mesenchymal cells into osteoblast-like cells [24]. Changes of the mitral ring, which extend towards valve leaflets, are particularly frequent [25, 26]. In this study mitral valve calcification in CKD patients was observed, but it was only slightly more frequent than in the control group. However, patients with CKD were more likely to have aortic valve calcification (study group 30.2 % vs. 0 % control group, *p* < 0.02). In another study, the joint prevalence of mitral or aortic valve calcification was 31 % in pre-dialysis patients, 50 % in dialysis patients and 12 % in control group (*p* = 0.001) [27]. In this study, contractile dysfunction, mitral valve calcification and aortic valve calcification in HD patients were significantly more frequent than in other groups of patients suffering from chronic kidney disease (*p* = 0.040; *p* = 0.044; *p* = 0.0008, respectively), which is consistent with results obtained in other studies [27]. Some studies demonstrated that the severity of vascular and valvular calcification in haemodialysis patients is associated with the incidence of cardiovascular complications and predicts cardiovascular mortality [25, 28]. Due to the fact that high frequency of cardiovascular disease cannot be explained only by the influence of traditional risk factors including smoking, hypertension, diabetes, disturbed lipid metabolism and aging there is a need to look for new mechanisms involved in its pathogenesis [22, 29, 30].

According to studies, in comparison to non-uremic serum, uremic serum increases the mineralization of vascular smooth muscle cells (VSMCs) and up-regulates the expression of Cbfa1/Runx2 and osteopontin (OPN), regardless of the serum P^2^ concentration [29, 31]. Bone-associated proteins such as fetuin A, osteoprotegerin (OPG), osteopontin (OPN) and MGP have been demonstrated to be expressed in atherosclerotic plaques and to participate in its calcification, while exogenous osteocalcin was shown to inhibit the process of calcification [32]. The level of osteocalcin (which is a non-collagenous, vitamin K-dependent protein produced by osteoblasts) is considered to be a non-invasive marker of osteoblast activity and bone formation [33]. In this study, no statistically significant differences in osteocalcin concentration between the control group and patients with chronic kidney disease were observed. However, it was found that the concentration of osteocalcin was highest in patients with stage I/II CKD and gradually decreased to reach its lowest value in patients with stage V/dialysis. Levels of osteocalcin in patients with CKD stage I-II and III were higher than in patients with higher CKD stages. Similar results were obtained in the study of Delmas et al. [34] who observed elevated levels of osteocalcin in patients with mild or moderate renal impairment. According to them, such results reflect the enhanced bone metabolism rather than decreased renal filtration.

Vitamin K-dependent MGP (matrix Gla-protein) is another important inhibitor of vascular calcification, which directly inhibits calcium precipitation and crystallization in the vessel wall and also plays a role in maintaining a normal phenotype of VSMCs and in preventing their differentiation into osteoblasts [29]. Although this study failed to reveal significant differences in the concentration of this protein between CKD patients and healthy volunteers, it was noted that MGP concentration decreased with worsening kidney function, which is consistent with other studies demonstrating significantly lower serum levels of uncarboxylated MGP (ucMGP) in dialyzed adult compared to healthy controls [35–37]. However, Schurgers et al. [36] observed that plasma levels of the inactive, dephosphorylated, uncarboxylated MGP (dp-ucMGP) levels increased progressively in the setting of CKD. Moreover, they reported an independent association between higher dp-ucMGP levels and aortic calcification as well as a limited relationship to overall mortality risk in CKD patients [38]. Osteoprotegerin deficiency is associated with vascular calcification through the inhibition of osteoclast differentiation and the modulation of bone resorption [29, 39]. Serum concentrations of osteoprotegerin seems to be a useful biomarker for early diagnosis of chronic kidney disease-mineral and bone disorder (CKD-MBD) [40]. In this study, osteoprotegerin concentrations in the control and study groups did not differ significantly. Osteoprotegerin levels were lowest in subjects with I/II stage CKD and gradually increased to reach its highest values in patients with stage V/dialysis. Morena et al. [41] also observed that a decline in renal function was associated with a significant increase in OPG. Omland et al. [42] demonstrated that raised levels of circulating OPG in patients with chronic kidney disease are associated with both aortic calcification and increased mortality. Moreover, in a study by Nascimento et al. [43], elevated OPG levels independently correlated with all-cause mortality and atherosclerosis assessed on the basis of increased IMT. However, it is still not known whether the increased levels of OPG levels reflects a protective, counter-regulatory effect or is associated with inflammatory processes which underlies the development of atherosclerosis [44, 45].

No significant differences in the levels of osteopontin and fetuin A between the control and study group were seen in this study. Fetuin A is a calcification inhibitor and reduced serum levels of this protein are associated with increased cardiovascular mortality in dialysis patients [24]. Westenfeld et al. [24] demonstrated that the co-existence of CKD, atherosclerotic vascular damage, hyperphosphatemia and fetuin-A deficiency is associated with significant increases in vascular calcification, almost exclusively intimal calcification of atheromatous lesions. Moreover, fetuin A deficiency in HD patients was found to be a predictor of inflammation-related cardiovascular and all-cause mortality, respectively [46, 47].

Osteopontin (OPN) has been recently identified as a component of human atherosclerotic plaque (in symptomatic carotid atherosclerosis [48] and in calcified coronary plaques [49]) implying a role for this protein in atherogenesis [50]. OPN protein was found to be abundant at calcification sites in human atherosclerotic plaques [49] and to be associated with carotid plaque vulnerability [51, 52], the presence and extent of coronary artery disease [50] in non-renal adult patients and myocardial remodeling, which might further influence ventricular function [53]. Barreto et al. [50] demonstrated elevated plasma OPN levels also in patients with chronic kidney disease, even at early stages, in comparison to healthy volunteers. They also reported that the positive association between plasma osteopontin level and clinical outcomes of CKD patients depended on their inflammatory status [50]. The lack of association between fetuin A and osteopontin in this study may be due to the relatively small sample size.

We also analysed the concentration of two matrix metalloproteinases (MMPs) and their inhibitors (TIMPs). Matrix metalloproteinases are endopeptidases responsible for the tissue remodeling and degradation of the extracellular matrix (ECM). The analysed MMPs −2 and −9 degrade type IV collagen, which is the main structural component of basement membrane [54]. Metalloproteinases are involved in atherogenesis and over-expression of MMP-2 and −9 has been observed within plaques [54, 55]. MMPs are able to damage fibrous cap of an atherosclerotic plaque thus making it unstable [56]. Matrix metalloproteinases (MMPs) production from macrophages could be enhanced by interferon (IFN)-γ from Th1 lymphocytes. IL-33 within IL-33/ST2 signaling pathway lowers serum levels of IFN-γ and prevents MMPs activation, retarding extracellular matrix destruction and plaque rupture [57]. Elevated serum levels of MMP-9 has been observed during the acute phase of myocardial infarction [58] with its maximum concentrations in the culprit coronary artery rather than systemic circulation [59]. In patients with non-ST segment elevation myocardial infarction (NSTEMI) the lower serum levels of IL-33 negatively correlated with MMP-9 (r = −0.461, *p* < 0.05) levels [56, 60]. Moreover, it has been suggested that elevated levels of MMP-2 and decreased concentration of MMP-9 are associated with the development of chronic kidney disease [61]. This study revealed significantly higher levels of MMP-2 in patients with chronic kidney disease in comparison to the control group. The lowest concentration of MMP-2 was seen in patients with CKD stage I/II and the highest in patients with stage IV and in those on dialysis. Our results are in accordance with the study of Pawlak et al. [62] who observed increased serum MMP-2 and also −9 in HD patients with a history of cardiovascular disease in comparison to patients without such history and control group. Chen et al. [63] demonstrated the role of MMP-2 and MMP-9 in arterial calcification. Moreover, they observed increased expression of MMP-2 and MMP-9 in the aorta of rats with progressive CKD as well as elevated serum activity of MMP-2. The over-expression of these two metalloproteinases was accompanied by the increased expression of transcription factor RUNX-2, which is thought to play an important role in the osteochondrocytic differentiation of VSMC and further in calcification [64].

Significant differences in concentration were also observed in metalloproteinase inhibitor TIMP-1. The highest concentration was observed in the control group, and the lowest in the group of patients with stage I/II CKD and in all CKD patients’ levels of TIMP-1 was lower than in the control group. Similarly to the results obtained by Musiał et al. [65] in the study of children with CKD, in this analysis, serum TIMP-1 concentrations increased in the late stages (II, IV) of renal failure which might be an anti-fibrotic response to extracellular matrix accumulation [66]. Some studies demonstrated that abundant TIMP-1 expression in the kidneys positively correlated with the extent of fibrosis [67–69]. However, in this study in all patients with CKD, TIMP-1 concentration was lower than in control group. Statistically significant results were found also for the MMP-2/TIMP-2 ratio, with the lowest values in the control group and the highest in patients with stage IV chronic kidney disease as well as in patients with stage V CKD and on dialysis. It has been suggested that CKD-associated MMP/TIMP imbalance disrupts the integrity of the extracellular matrix and leads to tissue remodeling, cells damage and matrix accumulation and further to atherosclerosis, renal fibrosis and enhanced cell migration to sites of inflammation [70]. Also, Rysz et al. [71] observed increased MMP-2/TIMP-2 ratio in HD patients compared with patients with CKD and controls. In contrast, in the study of Musiał et al. [70], MMP-2/TIMP-2 ratio was higher in CKD stages 2–3 vs. controls and thus they suggested that disturbances in MMP/TIMP balance are noticeable in early CKD, but as chronic kidney disease progresses it becomes corrected and stabilized. The discrepancies between studies results may be explained by differences in ethnicity of analysed populations, age, and CKD aetiology. The results of matrix metalloproteinases and their inhibitors analysis can be treated with caution due to the fact that their concentration may be influenced by used medications. According to Tayebjee MH [72] circulating MMP-9 levels are decreased while circulating TIMP-1 levels are increased after antihypertensive treatment. Moreover, it has been shown that nitroglycerin increases the expression and the activity of MMP-2, MMP-7 and MMP-9, and reduces TIMP-1 levels [73]. Medications such as calcium channel blockers (amlodipine, diltiazem), angiotensin II and angiotensin converting enzyme (ACE) inhibitors affects the activity of MMPs, not affecting its expression [74–76].

Multivariable analysis of comorbidities and protein concentrations demonstrated that the presence of heart failure was associated with the presence of chronic kidney disease. Also in the study of Heywood et al. [77] there was a relationship between the prevalence of coronary artery disease and worsening kidney function. However, due to the fact that heart failure and CKD share common risk factors it is often difficult to assess whether CKD in heart failure is prevalent or incident CKD, or rather a manifestation of cardio-renal syndrome [78, 79]. This multivariable analysis also revealed association between heart failure and osteopontin level. Also López et al. [80] found that plasma OPN was abnormally increased in patients with HF of hypertensive origin. Moreover, multivariable analysis including demographic, clinical and biochemical parameters indicated that osteopontin could be an independent predictor of death (hazard ratio 2.3, 95 % confidence interval 1.4 to 3.5, *P* < 0.001) and that it might be useful as a novel prognostic biomarker in patients with chronic heart failure [81].

In this study, heart failure was also associated with MMP-2, GM-CSF and atherosclerotic index of plasma (AIP). The association between MMPs and heart failure may be due the fact that metalloproteinases influences the process of atherosclerotic lesion formation due to intensified migration and proliferation of vascular smooth muscle cells in the intimal space, as well as the degradation of the fibrous cap of vulnerable atherosclerotic lesions [82]. According to Dobiasova et al. [83] atherogenic index plasma (AIP) (which is the logarithm of the plasma triglyceride level to high-density lipoprotein cholesterol) correlates with LDL particle size. A strong relationship between increased AIP high levels of small-dense LDL particles was demonstrated. The study of haemodialysis patients revealed that although in this group LDL levels were lower than in the controls, AIP ratio was higher, which may suggest that the size of LDL-c particles is of higher importance than their concentration in rapidly progressing atherosclerosis in ESRD. AIP was also suggested to be a subclinical atherosclerosis marker [84].

Moreover, in our multivariate analysis there was also relationship between phosphates (Pi) above normal level and osteoprotegerin (OPG). The study of paediatric patients with chronic kidney disease [85] provided plausible explanation for the association observed in our study. Siomou et al. [85] demonstrated a positive correlation between OPG levels and fibroblast growth factor-23 (FGF-23) levels which was not independent of serum Pi concentrations, which as they suggested may indicates possible compensatory reaction of OPG synthesis in response to increased Pi levels. In case of elevated serum phosphate levels, FGF-23 is secreted from the bone and it acts on the kidney to induce phosphaturia in order to maintain phosphate homeostasis [85].

Additionally, in our study the presence of hypertension was associated with chronic kidney disease, while lipid disorders with non-HDL cholesterol, AIP (high risk) and total cholesterol.

Both associations are not a new finding. It is commonly known that the relationship between hypertension and CKD is of cyclic nature. On the one hand, uncontrolled hypertension is an important risk factor for the development of CKD and is the second leading cause of ESRD [5], but on the other hand chronic kidney disease is one of the most common causes of secondary hypertension with prevalence increasing progressively with the severity of CKD [86]. Also the relationship between lipid disorders and non-HDL cholesterol, total cholesterol and AIP (high risk) is not surprising. Numerous studies indicate abnormalities in lipid metabolism in patients with all stages of chronic kidney disease (CKD) [87–89]. These abnormalities refer to all lipoprotein classes and depend on the degree of renal impairment, the aetiology of primary disease and dialysis method [90]. In CKD and dialysis patients, hypertriglyceridemia seems to be the most common form of dyslipidemia. [91]. All lipid abnormalities observed in chronic kidney disease including also diminished serum apoA-1 and high-density lipoprotein (HDL) concentrations, defective HDL maturation and its impaired antioxidant, anti-inflammatory and reverse cholesterol transport properties as well as compromised clearance of very low-density lipoprotein and chylomicrons in addition to oxidative stress are associated with increased risk of atherosclerosis in this group of patients [92]. Thus, it is not surprising that high risk AIP was observed in CKD patients in this study. Since AIP, as it was mentioned above, is the logarithm of plasma triglycerides to high-density lipoprotein cholesterol, its relationship with lipid disorders is not surprising. Moreover, it should be kept in mind that the size of LDL-c particles (and perhaps HDL particles) may be more important than their concentration.

We also analysed the relationship between protein concentration and various biochemical markers. An association between higher fetuin A concentration and increased level of alkaline phosphatase (ALP) was noted. We did not find any study observing a similar correlation. Serum ALP is a marker of bone turnover used to monitor the metabolic bone disease associated with renal insufficiency [93]. Experimental studies revealed that alkaline phosphatase might promote vascular calcification [94]. A high level of fetuin A coexisting with increased concentration of ALP may act as a defence mechanism against calcification. However, fetuin-A-mediated inhibition is overwhelmed in CKD and especially in CKD/HD [95]. Our study also revealed an associations between lower levels of osteocalcin and both elevated serum inorganic P and increased levels of urea as well as between higher levels of osteoprotegerin and increased concentrations of inorganic P. In patients with CKD, it is well established that hyperphosphatemia is associated with the development of vascular calcification [27, 62, 96]. In the past, vascular calcification induced by high serum phosphate was explained by simply exceeding (Ca_2_-P^2^) solubility, resulting in the precipitation of calcium phosphate. However, recent studies have demonstrated that high extracellular phosphate levels induce the transformation of VSMCs into osteoblast-like cells, which suggests that vascular calcification is an active process. Moreover, elevated extracellular phosphate levels are associated with the induction of Cbfa1/Runx2, a specific transcription factor for osteoblastic differentiation and the increase in bone-associated proteins such as osteocalcin, osteopontin and alkaline phosphatase (ALP) [27, 97, 98].

In this study, also a correlation between higher levels of metalloproteinase MMP-2 as well as higher values of MMP-2/TIMP-2 ratio and the prevalence of arterial hypertension was observed. The study of Chung et al. [64] demonstrated a correlation of MMP-2 with arterial stiffness in CKD patients. Moreover, Odenbach et al. showed that MMP-2 inhibition attenuated Ang II-induced hypertension [98]. According to Pawlak et al. [99], MMP-2/TIMP-2 ratio was higher in peritoneal dialysis (CAPD) patients with cardiovascular disease than in patients without CAD and healthy controls, and it was associated with quinolinic acid (QA) levels and increased oxidative status, suggesting the connection between kynurenine (KYN) pathway activation, arterial remodelling and CVD prevalence in uremic patients. Finally, in our study a statistically significant correlation between MMP-2/TIMP-2 and elevated values of CRP was seen. We found no studies confirming this correlation. However, in a study by Rysz et al. CRP was positively correlated with MMP-9 and MMP-9/TIMP-1 ratio in haemodialysis patients and patients with CKD [69].

## Conclusions

This study shows that the prevalence of ventricular hypertrophy and diastolic dysfunction in CKD patients is high. Moreover, in patients with CKD stage V/dialysis the E/E’ ratio was significantly higher than in other groups and tricuspid insufficiency was significantly more prevalent. Additionally, contractile dysfunction, mitral valve calcification and aortic valve calcification in HD patients were significantly more frequent than in other groups of patients suffering from chronic kidney disease. In this study, significantly increased levels of MMP-2, MMP-2/TIMP-2 ratio and lower levels of TIMP-1 were observed, suggesting that these factors may be involved in the pathogenesis of atherosclerosis in patients with CKD. Analysis of the levels of proteins associated with bone metabolism did not show statistically significant differences in the level of the analysed proteins between the healthy group and patients with chronic renal failure. Lack of significant correlations between bone-associated proteins could be due to the fairly small size of groups. In patients with CKD hypertrophy and calcification of the aortic valve were observed more frequently, which may suggest the reasons for increased cardiovascular risk in CKD patients.

### Limitations

Our study has some limitations. The number of participants included to the study is relatively small (80 patients with CKD and 24 healthy volunteers) due to the fact that it was a preliminary study. In this study, there may be a selection bias toward patients with associated disorders that might influence laboratory results due to the fact that patients for both control and study group were recruited among hospitalized persons. Study and control groups differ in age due to the difficulty to find healthy people aged 60–70 years to match study group. There are also differences in other demographic data such as sex, diabetes mellitus and hypertension between groups. Another limitation of this study is its cross-sectional design.
