# Erratum to: Markers of increased atherosclerotic risk in patients with chronic kidney disease: a preliminary study

**DOI:** 10.1186/s12944-016-0230-7

**Published:** 2016-03-22

**Authors:** Anna Gluba-Brzózka, Marta Michalska-Kasiczak, Beata Franczyk, Marek Nocuń, Peter P. Toth, Maciej Banach, Jacek Rysz

**Affiliations:** Department of Nephrology, Hypertension and Family Medicine, WAM University Hospital of Lodz, Poland, Żeromskiego 113, 90-549 Łódź, Poland; Department of Hypertension, Medical University of Lodz, Poland, Żeromskiego 113, 90-549 Łódź, Poland; Nofer Institute of Occupational Medicine, Lodz, Poland, Św. Teresy od Dzieciątka Jezus 8, 91-348 Łódź, Poland; Preventive Cardiology, CGH Medical Center, Sterling, IL USA; The Johns Hopkins Ciccarone Center for the Prevention of Heart Disease, Baltimore, MD USA; Healthy Aging Research Center, Medical University of Lodz, Lodz, Poland

## Erratum

After publication of the original article [1], it came to the authors’ attention that the middle initial of Peter P. Toth had been mistakenly omitted. The author list of the original article has been updated to include Dr Toth’s middle initial, which is also presented in this erratum.
